# Synopsis of a clinical practice guideline for pancreatic ductal adenocarcinoma with peritoneal dissemination in Japan; Japan Peritoneal Malignancy Study Group

**DOI:** 10.1002/jhbp.1085

**Published:** 2022-01-07

**Authors:** Sohei Satoi, Naminatsu Takahara, Tsutomu Fujii, Hiroyuki Isayama, Suguru Yamada, Yasushi Tsuji, Hideyo Miyato, Hironori Yamaguchi, Tomohisa Yamamoto, Daisuke Hashimoto, So Yamaki, Yousuke Nakai, Kei Saito, Hayato Baba, Toru Watanabe, Shigeto Ishii, Masamichi Hayashi, Keisuke Kurimoto, Hideaki Shimada, Joji Kitayama

**Affiliations:** ^1^ Department of Surgery Kansai Medical University Hirakata Japan; ^2^ Division of Surgical Oncology University of Colorado Anschutz Medical Campus Aurora Colorado USA; ^3^ Department of Gastroenterology Graduate School of Medicine The University of Tokyo Tokyo Japan; ^4^ Department of Surgery and Science Faculty of Medicine Academic Assembly University of Toyama Toyama Japan; ^5^ Department of Gastroenterology Graduate School of Medicine Juntendo University Tokyo Japan; ^6^ Department of Surgery Nagoya Central Hospital Nagoya Japan; ^7^ Department of Medical Oncology Tonan Hospital Sapporo Japan; ^8^ Department of Gastrointestinal Surgery Jichi Medical University Shimotsuke Japan; ^9^ Department of Clinical Oncology Jichi Medical University Shimotsuke Japan; ^10^ Department of Surgery II Nagoya University Graduate School of Medicine Nagoya Japan; ^11^ Department of Gastroenterological Surgery and Clinical Oncology Toho University Graduate School of Medicine Tokyo Japan

**Keywords:** guideline, pancreatic ductal adenocarcinoma, peritoneal dissemination

## Abstract

Patients with pancreatic ductal adenocarcinoma (PDAC) with peritoneal dissemination have a dismal prognosis because discontinuation of systemic chemotherapy is required for massive ascites or poor performance status. The natural history, diagnosis and treatment of PDAC with peritoneal dissemination have not been fully investigated. We systematically reviewed published information on the clinical diagnosis and treatment of PDAC with peritoneal dissemination using the PubMed database (2000‐2020) and provided recommendations in response to clinical questions. This guideline was created according to the "Minds Clinical Practice Guideline Development Guide 2017". The literature quality and body of evidence were evaluated with the GRADE System and classified into four levels (“strong”, “medium”, “weak”, “very weak”). The strength of each final recommendation was decided by a vote of committee members based on the GRADE Grid method. These guidelines address three subjects: diagnostic, chemotherapeutic, and surgical approaches. They include nine clinical questions and statements with recommendation strengths, evidence levels, and agreement rates, in addition to one “column”. This is the English synopsis of the 2021 Japanese clinical practice guideline for PDAC with peritoneal dissemination. It summarizes the clinical evidence for the diagnosis and treatment of PDAC with peritoneal dissemination and provides future perspectives.

## Abstract 2

**Highlight**

On behalf of the Japan Peritoneal Malignancy Study Group, Satoi and colleagues present an English synopsis of the 2021 Japanese clinical practice guidelines for pancreatic ductal adenocarcinoma with peritoneal dissemination. It summarizes the clinical evidence for the diagnosis and treatment of pancreatic ductal adenocarcinoma with peritoneal dissemination and provides future perspectives.

## 1 INTRODUCTION

The incidence of pancreatic ductal adenocarcinoma (PDAC) in Japan is predicted to increase continuously from 42 800 new cases in 2020 to 48 040 new cases in 2030.^1^ PDAC is now ranked as the fourth most common cause of cancer death, following lung, colon, and gastric cancer, and the 5‐year overall survival rate is 8.9%, which is significantly worse than 72% in colon cancer and 67.5% in gastric cancer diagnosed from 2009 to 2011.^1^ Poor survival is considered to result from the fact that 70%‐80% of patients with PDAC have unresectable disease at first presentation. Unresectable PDAC occurs with locally advanced and metastatic disease. The majority of distant organ metastasis occurs in the liver, peritoneum and lung.

New chemotherapeutic regimens, such as fluorouracil plus leucovorin, irinotecan, oxaliplatin (FOLFIRINOX)^2^ or gemcitabine + nab‐paclitaxel^3^ improve median survival time (MST) to 11 months or 8.5 months, respectively, in patients with metastatic PDAC. Since a large‐scale study did not contain enough patients with peritoneal dissemination to enable statistical analysis, the clinical efficacy of systemic chemotherapy has not been fully elucidated in this patient subpopulation.

Peritoneal dissemination is classified as macroscopic, appearing as peritoneal deposits, and microscopic, presenting as cancer cells in ascites or in peritoneal lavage (CY^+^). In a population‐based study from the Netherlands,^4^ between 1995 and 2009, patients diagnosed with peritoneal dissemination represented 9.1% of 2924 patients with PDAC. In updated data from 2005 to 2015, peritoneal dissemination was diagnosed in 7.7% of 19 098 patients with PDAC, and their MST was only 3.4 months (pancreatic head tumor), 2.3 months (pancreatic body tumor), and 2.2 months (pancreatic tail tumor).^5^ Takahara et al.^6^ reported the clinical outcomes of systemic chemotherapy in patients with malignant ascites. Overall survival (OS) in 21 patients with performance status 0‐2 was significantly better than that in 35 patients receiving best supportive care alone (124 vs 50 days, *P* < .01). A multivariate analysis revealed that chemotherapy was a significant independent prognostic factor.

During disease progression in PDAC with peritoneal dissemination, many patients frequently suffer from concomitant symptoms, such as abdominal fullness, appetite loss, abdominal pain, constipation, and/or oliguria, due to massive ascites, obstructive ileus or urethral obstruction. The presence of these symptoms can be associated with poor performance and nutritional status, resulting in less opportunity to receive systemic chemotherapy.^7^ Thus, chemotherapy for patients with PDAC with peritoneal dissemination requires relief of specific symptoms and prognostic improvement.

The purpose of this guideline is to provide information on the management of PDAC with or suspicious for peritoneal dissemination. Our aims are to improve clinical practice, patient quality of life and survival. In this guideline, we systematically review the diagnostic and treatment approaches used in patients with macroscopic and microscopic peritoneal dissemination to clarify the current status of this disease entity in the real world. Since the quantity and quality of evidence are less for PDAC with peritoneal dissemination, clinical and practical guidance for diagnostic and therapeutic approaches are required for better clinical and practical management. This guideline represents the most standard one currently available, reflecting the national medical insurance system in Japan. This is the English synopsis of the 2021 Japanese practice guidelines for PDAC with peritoneal dissemination.^8^

## 2 METHODS

We tried to organize the information on various treatments for PDAC with peritoneal dissemination and to clarify the degree of recommendation for clinical questions (CQs), with the aims of producing a good social environment where medical professionals and patients understand the treatment plan for peritoneal dissemination well, and of providing high‐quality medical care. Off‐label drug use in Japan was clarified in the CQs and statements. The guideline committee consisted of gastroenterologists, surgeons, endoscopists, medical oncologist, and palliative care physicians.

This guideline was created according to the "Minds Clinical Practice Guideline Development Guide 2017". A systematic review was performed with related keywords for each CQ, and related papers were collected comprehensively using the PubMed database (2000‐2020). For some CQs with a small number of relevant articles, additional papers in Igaku Chuo Zasshi (ICHUSHI), a Japanese bibliographic database, as well as ASCO Proceedings were selected by a manual search. The evidence level was indicated by the volume of individual papers related to the critical outcomes included within the CQs and divided into groups by study design and quality. The literature level and body of evidence were evaluated in reference to the GRADE System and ultimately classified into four levels: "strong", "medium", "weak", and "very weak". Based on the results, draft recommendation statements and the strength of the recommendations were evaluated at a consensus meeting of the Guideline Committee. After discussion, the balance between the benefits and harms, patient value and hopes, cost effectiveness, and feasibility of being performed at general facilities nationwide were considered, and the strength of the final recommendation was decided by a vote of committee members based on the GRADE Grid method. We selected one of the following five options for the vote and recommendation, as follows: (1) Strong “For” intervention, (2) Weak “For” intervention, (3) Weak “Against” intervention, (4) Strong “Against” intervention, (5) Not graded. With one vote, if 70% or more of the votes were obtained for any of options (1) to (5), the decision was considered final. If (1) + (2) exceeded 50% and (3) + (4) was 20% or lower, the decision was “weakly recommend to perform.” If (3) + (4) exceeded 50% and (1) + (2) was 20% or lower, the decision was “weakly recommend not to perform.” If these criteria could not be met, then the results were disclosed and discussed and the members re‐voted. If no agreement was reached, the decision of "(5) Not graded” was selected.

Two members of an external evaluation committee had evaluated these guidelines independently. Furthermore, the member of guidelines evaluation committee of Japanese Society of clinical oncology evaluated these guidelines according to the GRADE II. Subsequently, public comments were collected on the web page of Japanese Society of clinical oncology between March 1 and March 31, 2021.

This guideline is targeted to medical doctors who manage patients with PDAC, other medical doctors and staff, patients and their families and any other individuals interested in PDAC with peritoneal dissemination to provide information on the current management of PDAC with peritoneal dissemination. This guideline will be revised 3 years after publication.

This article is an English translation of a part of the Japanese version of the clinical practice guideline for pancreatic ductal adenocarcinoma with peritoneal dissemination which was obtained the acceptance of secondary publication by KANEHARA and CO., LTD.^8^

## 3 RESULTS

### 3.1 Diagnostic approach to peritoneal dissemination

The natural history of peritoneal dissemination remains unclear because of the difficulty of early diagnosis. Clinically, it is common to make a diagnosis based on the presence of massive ascites, multiple peritoneal nodules and/or omental cake with a high level of CA19‐9, which means an intractable stage of PDAC with peritoneal dissemination. Although abdominal ultrasonography, contrast‐enhanced CT imaging (CE‐CT), magnetic resonance imaging (MRI), endoscopic ultrasound (EUS), and positron emission tomography (PET)‐CT have been utilized, CT imaging is limited in the detection of intestinal or mesenterial deposits and may provide an underestimation of disease volume.^9^, ^10^ On comparing the diagnostic role of imaging studies with intra‐operative findings, the sensitivity of PET, CT, or PET/CT was 46%‐63%, 80%‐84%, or 85%‐89%, and the specificity of those was 89%‐95%, 77%‐88%, or 85%‐90%, respectively. Subsequently, PET‐CT was reported to reflect the extent of peritoneal dissemination well.^11^, ^12^ Aherne et al.^13^ reviewed the role of imaging studies by radiologists: (1) Ultrasound may be used for the initial identification of patients with ascites, peritoneal deposits, pelvic masses, or bowel distention, and for percutaneous biopsy with the use of real‐time imaging. (2) CE‐CT is the current reference standard for staging and is used to calculate the CT peritoneal cancer index score. (3) MRI and PET/CT have a role in evaluating complex cases or characterizing cases with equivocal findings in an effort to reduce the need for invasive procedures. However, In the future, PET/CT may become the first‐line staging tool for patients with peritoneal dissemination.

A specific tumor marker for peritoneal dissemination is still under investigation. Although CA19‐9, CEA, and Dupan‐2 are generally considered useful tumor markers even in these patients, the cut‐off level for the presence of peritoneal dissemination has not been fully investigated.

Some articles have reported that staging laparoscopy clearly detected the presence of latent distant organ metastasis in 20%‐40% of patients with radiographically defined locally advanced PDAC.^7^, ^14^, ^15^, ^16^, ^17^, ^18^, ^19^ Ta et al^20^ reported in a meta‐analysis that with staging laparoscopy, occult peritoneal dissemination was found in 19% of 367 patients with PDAC. Karabicak et al.^14^ reported that staging laparoscopy diagnosed microscopic peritoneal dissemination in 23% and macroscopic peritoneal dissemination in 19% of 110 patients with radiographically defined, unresectable, locally advanced PDAC. They suggested that PDAC located in the pancreas body‐tail and tumor size >42 mm were risk factors for peritoneal dissemination, and 65.4% of patients with these factors had peritoneal dissemination. Takadate et al.^21^ demonstrated the presence of microscopic peritoneal dissemination during staging laparoscopy in 24% (n = 10) of patients with resectable disease (n = 42), 22% (n = 11) of patients with borderline resectable disease (n = 49), and 38% (n = 21) of patients with locally advanced disease (n = 55). Moreover, staging laparoscopy showed the presence of macroscopic peritoneal dissemination during staging laparoscopy in 0% of patients with resectable disease, 6% (n = 3) of patients with borderline resectable disease, and 11% (n = 6) of patients with locally advanced disease. Thus, the incidence of peritoneal dissemination increased according to the resectability status, and therefore, staging laparoscopy is mandatory for more accurate diagnosis of peritoneal dissemination, because imaging studies have limitations for detecting minute peritoneal dissemination.

### 3.2 Therapeutic approach to peritoneal dissemination (Chemotherapy and surgery)

Even if systemic chemotherapy is implemented as a standard treatment, its continuation is difficult due to cancer‐associated symptoms, such as ascites or malnutrition. Systemically administered drug delivery to the peritoneum is limited. Therefore, the MST in patients with peritoneal dissemination in population‐based studies of malignant ascites was reported to range from 6 weeks to 3 or 4 months,^4^, ^5^, ^6^ which was clearly worse than that for other sites of metastasis, such as liver or lung. In contrast, the MST in patients with occult peritoneal dissemination diagnosed by staging laparoscopy was approximately 7 months.^7^, ^19^ There is an obvious gap in MST between patients with peritoneal dissemination diagnosed by clinical manifestation and staging laparoscopy. Establishment of disease staging in PDAC with peritoneal dissemination is required.

The majority of patients with peritoneal dissemination diagnosed by staging laparoscopy frequently develop ascites during systemic chemotherapy, resulting in a shorter duration of first‐line chemotherapy and a lower proportion of second‐line chemotherapy compared with patients with liver metastasis or locally advanced disease.^7^ Therefore, important goals of treatment would be to control the development of ascites and to improve survival in patients with PDAC with peritoneal dissemination who have poor quality of life and a dismal prognosis. Compared with systemic chemotherapy, intraperitoneal chemotherapy appears to be advantageous for the treatment of peritoneal dissemination due to the high drug concentration achieved in the peritoneal cavity to directly contact tumor nodules.^22^, ^23^, ^24^, ^25^, ^26^, ^27^, ^28^, ^29^ Although hyperthermic intraperitoneal chemotherapy (HIPEC),^30^, ^31^ and pressurized intraperitoneal aerosol chemotherapy (PIPAC)^32^, ^33^ have also been implemented in patients with PDAC with peritoneal dissemination, the clinical efficacy of these options in patients with PDAC is still under investigation. Intraperitoneal chemotherapy with paclitaxel provided a better MST of 14‐16 months in a cohort of patients with occult peritoneal dissemination and of 28 months or longer in patients who underwent conversion surgery in phase II studies.^26^, ^27^ A phase III multicenter randomized clinical trial (RCT) is ongoing (UMIN000027229/jRCTs051180199).

The 2019 clinical practice guidelines for pancreatic cancer from the Japan Pancreas Society revealed that it was not clear whether surgery was indicated for patients with positive peritoneal lavage cytology.^34^ The clinical efficacy of surgical resection in patients with microscopic peritoneal dissemination and resectable disease remains controversial.^35^, ^36^ A recent large‐scale retrospective study reported that positive peritoneal washing cytology was a significant independent prognostic factor in patients with PDAC who underwent surgical resection, and curative resection followed by adjuvant chemotherapy might contribute to the long‐term prognosis of patients with positive cytology status.^37^

This guideline addresses three subjects (diagnosis, chemotherapy and surgery) consisting of nine clinical questions (blood tests, imaging studies, abdominal paracentesis, staging laparoscopy, systemic chemotherapy, intraperitoneal chemotherapy, HIPEC, conversion surgery, and surgery for P0CY1), and one “column” of PIPAC. It contains statements corresponding to clinical questions with recommendation strengths, evidence levels, and agreement rates.

#### 3.2.1 Diagnosis

##### CQ1: Are blood tests (tumor markers) recommended for the diagnosis of peritoneal dissemination?

*Statement*: Measurement of tumor markers such as serum cancer antigen (CA) 19‐9 is weakly recommended.

[Weak recommendation, Evidence level C, proportion of agreement (8/8, 100%)].

*Future perspective*: The diagnostic value of tumor markers has not been fully investigated. However, a high tumor marker level may reflect the presence of occult distant organ metastasis, such as peritoneal dissemination, in clinical practice. The optimal cut‐off level of each tumor marker should be investigated for diagnosing peritoneal dissemination, and a prospective validation study will be required.

##### CQ2: Are imaging studies recommended for the diagnosis of peritoneal dissemination?

*Statement*: It is weakly recommended to perform imaging studies (contrast‐enhanced multi‐detector row CT, magnetic resonance imaging, endoscopic ultrasound, and positron emission tomography‐CT) for the diagnosis of peritoneal dissemination.

[Weak recommendation, Evidence level C, proportion of agreement (8/8, 100%)].

*Future perspective*: The diagnosis and definition of peritoneal dissemination have not been standardized in the literature; macroscopic or microscopic examination under staging laparoscopy or open laparotomy; peritoneal cytology; imaging studies with clinical features, etc. Some articles have reported a high incidence of occult peritoneal dissemination diagnosed using staging laparoscopy or open laparotomy in patients with radiographically defined, locally advanced PDAC. Imaging studies may underestimate the presence of peritoneal dissemination. Well‐designed clinical studies to assess the diagnostic value of imaging studies compared with macroscopic and microscopic findings are required.

##### CQ3: Is abdominal paracentesis recommended for the diagnosis of peritoneal dissemination?

*Statement*: It is weakly recommended to perform abdominal paracentesis for the diagnosis of peritoneal dissemination.

[Weak recommendation, Evidence level C, proportion of agreement (8/8, 100%)].

*Future perspective*: Positive peritoneal cytology (microscopic peritoneal dissemination) is considered to indicate the presence of peritoneal dissemination in patients with ascites. However, the presence of microscopic peritoneal dissemination may not be synonymous with macroscopic dissemination. The gold standard for the diagnosis of macroscopic peritoneal dissemination is surgical exploration; however, it is not always indicated in patients with advanced PDAC. Thus, the clinical course as well as the association between microscopic and macroscopic peritoneal dissemination remain unclear and should be investigated.

##### CQ4: Is staging laparoscopy recommended in patients suspicious for peritoneal dissemination?

*Statement*: Staging laparoscopy is useful for diagnosing peritoneal dissemination when it is difficult to assess with imaging devices. It is weakly recommended to perform staging laparoscopy in patients suspicious for peritoneal dissemination under appropriate selection of patients who have a planned open surgery.

[Weak recommendation, Evidence level C, proportion of agreement (7/8, 88%)].

*Summary*: The rate of peritoneal dissemination was reported to be 0.74%‐8% in patients of resectable lesions, 12.1% in patients of borderline resectable lesions, and 19.0%‐47.8% in patients of locally advanced lesions. Thus, patients with locally advanced lesions could be candidates for high‐risk peritoneal dissemination, although evidence is limited.^7^, ^17^, ^18^

*Future perspective*: Staging laparoscopy for patients who intend to undergo surgical resection is useful for improving the diagnostic value and avoiding an unnecessary open laparotomy. Considering cost effectiveness and peri‐operative complications, appropriate patient selection is mandatory for performing staging laparotomy. Therefore, determination of high‐risk groups for peritoneal dissemination should be investigated. There is less evidence for risk factors for peritoneal dissemination, and they may be identified according to the resectability status of PDAC. A meta‐analysis and a validation study will be required for investigating high‐risk groups.

#### 3.2.2 Chemotherapy

##### CQ5: Is systemic chemotherapy recommended for patients with peritoneal dissemination?

*Statement*: The presence of peritoneal dissemination is frequently associated with the development of hydronephrosis or intestinal obstruction, worsening the general condition rapidly, and therefore, systemic chemotherapy is recommended, fully considering the patient's condition.

[Strong recommendation, Evidence level B, proportion of agreement (6/8, 75%)].

*Future perspective*: No prospective studies have investigated the clinical effects of systemic chemotherapy for PDAC with peritoneal dissemination. Pharmacokinetic studies revealed that anticancer drugs administered systemically do not retain a sufficient drug concentration in the peritoneal cavity.

Clinical features of patients with peritoneal dissemination are unique and different from those with other distant metastases. To establish an optimal treatment approach for PDAC with peritoneal dissemination, it is necessary to verify the role of systemic chemotherapy in well‐designed clinical trials targeting patients with peritoneal dissemination.

##### CQ6: Is intraperitoneal chemotherapy recommended in patients with peritoneal dissemination?

*Statement*: Intraperitoneal chemotherapy is weakly recommended in patients with peritoneal dissemination who do not have a large amount of ascites (off‐label use).

[Weak recommendation, Evidence level C, proportion of agreement (8/8, 100%)].

*Summary*: Some studies of intraperitoneal paclitaxel and systemic chemotherapy showed promising results in terms of response rate, survival time and rate of conversion to surgical resection in patients with malignant ascites or occult peritoneal dissemination.^26^, ^27^, ^28^, ^29^ Although this combined chemotherapy may be effective in patients with no other distant metastasis, less ascites, and good performance status, the therapeutic effect may be limited to patients with other organ metastases and poor performance status. Considering the requirement of placement of an intraperitoneal access port and the incidence of adverse events associated with combined chemotherapy, from the point of invasiveness, a comparative study of good quality is needed.

*Future perspective*: In the published literature, there were roughly two treatment target groups (occult peritoneal dissemination and malignant ascites), and there was no standard for separating them. Thus, it is necessary to establish a clinical staging system for peritoneal dissemination. The additional effect of the combined use of intra‐abdominal chemotherapy and systemic chemotherapy should be verified in a comparative study with standard treatment (systemic chemotherapy). It is also necessary to consider the most appropriate systemic chemotherapy regimen that should be used in combination with intra‐abdominal chemotherapy.

##### CQ7: Are cytoreductive surgery and HIPEC recommended in patients with peritoneal dissemination?

*Statement*: It is weakly recommended that cytoreductive surgery and HIPEC should not be performed in patients with peritoneal dissemination.

[Weak recommendation, Evidence level D, proportion of agreement (8/8, 100%)].

*Summary*: No clinical trials have been conducted to evaluate the effects of cytoreductive surgery and HIPEC for PDAC with peritoneal dissemination; only case reports and case accumulation studies have been published.^38^, ^39^, ^40^ All of these contain a small number of cases (8 or less), and the drugs used for HIPEC vary, such as cisplatin, 5‐FU, gemcitabine, and mitomycin C, so efficacy cannot be discussed. Regarding safety, Faruma et al.^38^ reported a perioperative complication rate of 55.6% and a treatment‐related mortality rate of 5.6% in a case accumulation study of 18 patients that included seven patients with PDAC with peritoneal dissemination. In addition, in a case accumulation study by Tentes et al,^39^ treatment‐related deaths were reported in 2 of 8 cases.

*Benefits*: There is no clear evidence of extended survival or improved quality of life.

*Harm*: Perioperative complications, treatment‐related deaths.

*Future perspective*: Although many clinical trials of cytoreductive surgery and HIPEC have been conducted in patients with peritoneal dissemination of colorectal cancer and gastric cancer, there is currently little evidence of their efficacy and safety in PDAC with peritoneal dissemination. Evidence is needed for multidisciplinary treatment that combines surgery, systemic chemotherapy, and intraperitoneal therapy for PDAC with peritoneal dissemination, which is considered to be more aggressive.

#### 3.2.3 Surgery

##### CQ8: Is conversion surgery recommended in patients with peritoneal dissemination who respond favorably to multimodal treatment?

*Statement*: It is weakly recommended that conversion surgery should be performed in patients whose peritoneal dissemination becomes undetectable macroscopically and microscopically.

[Weak recommendation, Evidence level C, proportion of agreement (8/8, 100%)].

*Summary*: There is no definite evidence regarding the effectiveness of conversion surgery in patients with peritoneal dissemination. One phase II study showed a survival benefit in eight patients who underwent conversion surgery, relative to 25 patients who did not (MST: 27.8 vs 16.3 months, respectively; *P* = .0062).^26^ Another phase II study demonstrated improved survival in eight patients who underwent surgical resection relative to 38 patients who did not (MST: not reached vs 12.4 months, respectively; *P* = .004).^27^ Patients with favorable responses (disappearance of peritoneal dissemination, shrinkage of the advanced primary tumor, decreased tumor markers, good performance status, etc) to multimodal treatments may be candidates for conversion surgery, but a definite surgical indication remains controversial.

*Future perspective*: It is necessary to verify the clinical effects of multidisciplinary treatment for patients with peritoneal dissemination in a well‐designed study, since existing studies have reported clinical effects of multimodal treatment including intraperitoneal therapy in one‐arm single‐center or multi‐center prospective studies. In addition, it is difficult to plan an RCT to evaluate conversion surgery due to the heterogeneity of treatment. Results of a large‐scale study are awaited.

##### CQ9: Is surgical resection recommended in patients with resectable or borderline resectable PDAC and microscopic peritoneal dissemination?

*Statement*: It is weakly recommended that surgical resection with a surgery‐first approach should not be performed in patients with resectable PDAC and microscopic peritoneal dissemination.

[Weak recommendation, Evidence level C, proportion of agreement (8/8, 100%)].

*Future perspective*: There are no prospective studies or RCTs to address this CQ. Surgical resection following neo‐adjuvant therapy has become a mainstream treatment strategy for resectable or borderline resectable PDAC. Therefore, strong evidence must be constructed by designing a prospective study in patients with resectable or borderline resectable PDAC and microscopic peritoneal dissemination.

### 3.3 Column: Pressurized intraperitoneal aerosol chemotherapy

Pressurized intraperitoneal aerosol chemotherapy (PIPAC) is a new treatment approach for intraperitoneal chemotherapy.^41^ After insufflation of 12 mm Hg of capnoperitoneum at 37°C, two balloon trocars are placed. A nebulizer is connected to a high‐pressure injector and inserted into the abdomen through a trocar. A pressurized aerosol containing cisplatin in 150 mL of 0.9% NaCl is applied, immediately followed by doxorubicin in 50 mL 0.9% NaCl. The system is kept at steady‐state for 30 minutes (application time). PIPAC is repeated two to five times at various time intervals.

Intraperitoneal chemotherapy as a pressurized aerosol is considered to take advantage of the fact that applying an aerosol allows a homogeneous repartition of the substance within a closed space, resulting in a high drug concentration in the peritoneal tissues and low systemic exposure. Grass et al.^42^ reported in a systematic review that preclinical data suggested better distribution and higher tissue concentrations of chemotherapy agents with PIPAC compared with conventional intraperitoneal chemotherapy by lavage. They concluded that PIPAC was feasible, safe and well tolerated. Alyami et al.^43^ also reported in a systematic review that an objective clinical response and MST with PIPAC were 62%‐88% and 11‐14 months, respectively, in patients with ovarian cancer, 50%‐91% and 8‐15 months, respectively, in patients with gastric cancer, 71%‐86% and 16 months, respectively, in patients with colorectal cancer, and 67%‐75% and 27 months, respectively, in patients with peritoneal mesothelioma.

Some retrospective studies revealed that the use of PIPAC in patients with PDAC was safe and feasible and resulted in an MST in 9.2‐14 months.^32^, ^33^, ^44^, ^45^ These reports contained small numbers of patients with heterogenous backgrounds. The clinical effects of PIPAC and appropriate regimen selection must be investigated prospectively in the near future.

## 4 SUMMARY

Staging laparoscopy still has an important position in the accurate diagnosis of peritoneal dissemination because it can detect small nodules and enable pathological or cytological diagnosis of the small nodules or peritoneal washing fluid/ascites. Use of intraperitoneal chemotherapy is expected to prolong survival and lead to a high proportion of conversion surgery, even in patients with peritoneal dissemination, in phase II studies.^26^, ^27^ A phase III RCT is ongoing (UMIN000027229/jRCTs051180199). HIPEC appears to be harmful in Japanese patients, and a well‐designed clinical study is needed. Clinical trials for elucidating the clinical effects of PIPAC should also be conducted. The prognosis of patients with resectable/borderline resectable disease and positive peritoneal washing cytology is limited; therefore, implementation of multimodal therapy, but not upfront surgery, should be investigated. Since a lack of evidence remains regarding the natural history, definitive diagnosis, and appropriate treatment of PDAC with peritoneal dissemination, this clinical and practical guidance for diagnostic and therapeutic approaches is provided as the most standard guideline available at this time in Japan. Further clinical study should be conducted to provide a high level of good‐quality evidence to address the clinical questions raised. Sustainable efforts are warranted to support patients with PDAC and peritoneal dissemination who have poor quality of life and a high risk of death.

## CONFLICT OF INTEREST

Isayama H is supported by a grant and speaker fee from the Taiho Pharmaceutical Co., Ltd., and Yakult Honsha Company. Satoi S is supported by a funding for clinical research from Nihon Servier Co., Ltd. The other authors have no conflicts of interest. Nakai Y received a grant from Taiho Pharmaceutical Co, Ltd.

## Supporting information

Supplementary Material
