# Supplementary material for: Synopsis of a clinical practice guideline for pancreatic ductal adenocarcinoma with peritoneal dissemination in Japan; Japan Peritoneal Malignancy Study Group
Source: J Hepatobiliary Pancreat Sci. 2022 Jan 7;29(6):600–8. doi: 10.1002/jhbp.1085 (PMC9306579; doi:10.1002/jhbp.1085)
Supplement: Supplementary file 1 — Supplementary Material [file JHBP-29-600-s001.docx]

CQ 1.

1. Sharma C, Eltawil KM, Renfrew PD et al. Advances in diagnosis, treatment and palliation of pancreatic carcinoma: 1990-2010. World J Gastroenterol 2011; 17: 867-897.

2. Goggins M. Molecular markers of early pancreatic cancer. J Clin Oncol 2005; 23: 4524-4531.

3. Cwik G, Wallner G, Skoczylas T et al. Cancer antigens 19-9 and 125 in the differential diagnosis of pancreatic mass lesions. Arch Surg 2006; 141: 968-973; discussion 974.

4. Molina V, Visa L, Conill C et al. CA 19-9 in pancreatic cancer: retrospective evaluation of patients with suspicion of pancreatic cancer. Tumour Biol 2012; 33: 799-807.

5. Goonetilleke KS, Siriwardena AK. Systematic review of carbohydrate antigen (CA 19-9) as a biochemical marker in the diagnosis of pancreatic cancer. Eur J Surg Oncol 2007; 33: 266-270.

6. Maisey NR, Norman AR, Hill A et al. CA19-9 as a prognostic factor in inoperable pancreatic cancer: the implication for clinical trials. Br J Cancer 2005; 93: 740-743.

7. Humphris JL, Chang DK, Johns AL et al. The prognostic and predictive value of serum CA19.9 in pancreatic cancer. Ann Oncol 2012; 23: 1713-1722.

8. Alberghina N, Sanchez-Montes C, Tunon C et al. Endoscopic ultrasonography can avoid unnecessary laparotomies in patients with pancreatic adenocarcinoma and undetected peritoneal carcinomatosis. Pancreatology 2017; 17: 858-864.

9. Konigsrainer I, Zieker D, Symons S et al. Do patient- and tumor-related factors predict the peritoneal spread of pancreatic adenocarcinoma? Surg Today 2014; 44: 260-263.

10. Fujioka S, Misawa T, Okamoto T et al. Preoperative serum carcinoembryonic antigen and carbohydrate antigen 19-9 levels for the evaluation of curability and resectability in patients with pancreatic adenocarcinoma. J Hepatobiliary Pancreat Surg 2007; 14: 539-544.

11. Karabicak I, Satoi S, Yanagimoto H et al. Risk factors for latent distant organ metastasis detected by staging laparoscopy in patients with radiologically defined locally advanced pancreatic ductal adenocarcinoma. J Hepatobiliary Pancreat Sci 2016; 23: 750-755.

12. Alexakis N, Gomatos IP, Sbarounis S et al. High serum CA 19-9 but not tumor size should select patients for staging laparoscopy in radiological resectable pancreas head and peri-ampullary cancer. Eur J Surg Oncol 2015; 41: 265-269.

13. Fong ZV, Alvino DML, Fernandez-Del Castillo C et al. Reappraisal of Staging Laparoscopy for Patients with Pancreatic Adenocarcinoma: A Contemporary Analysis of 1001 Patients. Ann Surg Oncol 2017; 24: 3203-3211.

14. Liu X, Fu Y, Chen Q et al. Predictors of distant metastasis on exploration in patients with potentially resectable pancreatic cancer. BMC Gastroenterol 2018; 18: 168.

15. Maithel SK, Maloney S, Winston C et al. Preoperative CA 19-9 and the yield of staging laparoscopy in patients with radiographically resectable pancreatic adenocarcinoma. Ann Surg Oncol 2008; 15: 3512-3520.

16. Schlieman MG, Ho HS, Bold RJ. Utility of tumor markers in determining resectability of pancreatic cancer. Arch Surg 2003; 138: 951-955; discussion 955-956.

17. Takadate T, Morikawa T, Ishida M et al. Staging laparoscopy is mandatory for the treatment of pancreatic cancer to avoid missing radiologically negative metastases. Surg Today 2020.

18. Hata S, Sakamoto Y, Yamamoto Y et al. Prognostic impact of postoperative serum CA 19-9 levels in patients with resectable pancreatic cancer. Ann Surg Oncol 2012; 19: 636-641.

19. Takagi C, Kikuchi Y, Shirakawa H et al. Predictive Factors for Elevated Postoperative Carbohydrate Antigen 19-9 Levels in Patients With Resected Pancreatic Cancer. Anticancer Res 2019; 39: 3177-3183.

CQ2.

1. Jacquet P, Jelinek JS, Steves MA, et al. Evaluation of computed tomography in patients with peritoneal carcinomatosis. Cancer 1993;72:1631-6.

2. Fultz PJ, Jacobs CV, Hall WJ, et al. Ovarian cancer: comparison of observer performance for four methods of interpreting CT scans. Radiology 1999;212:401-10.

3. Coakley FV, Choi PH, Gougoutas CA, et al. Peritoneal metastases: detection with spiral CT in patients with ovarian cancer. Radiology 2002;223:495-9.

4. de Bree E, Koops W, Kroger R, et al. Peritoneal carcinomatosis from colorectal or appendiceal origin: correlation of preoperative CT with intraoperative findings and evaluation of interobserver agreement. J Surg Oncol 2004;86:64-73.

5. Laghi A, Bellini D, Rengo M, et al. Diagnostic performance of computed tomography and magnetic resonance imaging for detecting peritoneal metastases: systematic review and meta-analysis. Radiol Med 2017;122:1-15.

6. Marin D, Catalano C, Baski M, et al. 64-Section multi-detector row CT in the preoperative diagnosis of peritoneal carcinomatosis: correlation with histopathological findings. Abdom Imaging 2010;35:694-700.

7. Jensen CT, Vicens-Rodriguez RA, Wagner-Bartak NA, et al. Multidetector CT detection of peritoneal metastases: evaluation of sensitivity between standard 2.5 mm axial imaging and maximum-intensity-projection (MIP) reconstructions. Abdom Imaging 2015;40:2167-72.

8. Matsumoto I, Shirakawa S, Shinzeki M, et al. 18-Fluorodeoxyglucose positron emission tomography does not aid in diagnosis of pancreatic ductal adenocarcinoma. Clin Gastroenterol Hepatol 2013;11:712-8.

9. Karabicak I, Satoi S, Yanagimoto H, et al. Risk factors for latent distant organ metastasis detected by staging laparoscopy in patients with radiologically defined locally advanced pancreatic ductal adenocarcinoma. J Hepatobiliary Pancreat Sci 2016;23:750-755.

10. Liu RC, Traverso LW. Diagnostic laparoscopy improves staging of pancreatic cancer deemed locally unresectable by computed tomography. Surg Endosc 2005;19:638-42.

11. Zhang Y, Huang J, Chen M, et al. Preoperative vascular evaluation with computed tomography and magnetic resonance imaging for pancreatic cancer: a meta-analysis. Pancreatology 2012;12:227-33.

12. Treadwell JR, Zafar HM, Mitchell MD, et al. Imaging Tests for the Diagnosis and Staging of Pancreatic Adenocarcinoma: A Meta-Analysis. Pancreas 2016;45:789-95.

13. Bipat S, Phoa SS, van Delden OM, et al. Ultrasonography, computed tomography and magnetic resonance imaging for diagnosis and determining resectability of pancreatic adenocarcinoma: a meta-analysis. J Comput Assist Tomogr 2005;29:438-45.

14. Koelblinger C, Ba-Ssalamah A, Goetzinger P, et al. Gadobenate dimeglumine-enhanced 3.0-T MR imaging versus multiphasic 64-detector row CT: prospective evaluation in patients suspected of having pancreatic cancer. Radiology 2011;259:757-66.

15. Nawaz H, Fan CY, Kloke J, et al. Performance characteristics of endoscopic ultrasound in the staging of pancreatic cancer: a meta-analysis. JOP 2013;14:484-97.

16. Ho JM, Eysselein VE, Stabile BE. The value of endoscopic ultrasonography in predicting resectability and margins of resection for periampullary tumors. Am Surg 2008;74:1026-9.

17. Levy MJ, Abu Dayyeh BK, Fujii LL, et al. Detection of peritoneal carcinomatosis by EUS fine-needle aspiration: impact on staging and resectability (with videos). Gastrointest Endosc 2015;81:1215-24.

18. Alberghina N, Sanchez-Montes C, Tunon C, et al. Endoscopic ultrasonography can avoid unnecessary laparotomies in patients with pancreatic adenocarcinoma and undetected peritoneal carcinomatosis. Pancreatology 2017;17:858-864.

19. Mortensen MB, Fristrup C, Holm FS, et al. Prospective evaluation of patient tolerability, satisfaction with patient information, and complications in endoscopic ultrasonography. Endoscopy 2005;37:146-53.

CQ 3.

1. DeWitt J, Yu M, Al-Haddad MA, et al. Survival in patients with pancreatic cancer after the diagnosis of malignant ascites or liver metastases by EUS-FNA. Gastrointest Endosc 2010;71:260-5.

2. Takahara N, Isayama H, Nakai Y, et al. Pancreatic cancer with malignant ascites: clinical features and outcomes. Pancreas 2015;44:380-5.

3. Baretti M, Pulluri B, Tsai HL, et al. The Significance of Ascites in Patients With Pancreatic Ductal Adenocarcinoma: A Case-Control Study. Pancreas 2019;48:585-589.

4. Garrison RN, Galloway RH, Heuser LS. Mechanisms of malignant ascites production. J Surg Res 1987;42:126-32.

5. Cattau EL, Jr., Benjamin SB, Knuff TE, et al. The accuracy of the physical examination in the diagnosis of suspected ascites. JAMA 1982;247:1164-6.

6. Goldberg BB, Goodman GA, Clearfield HR. Evaluation of ascites by ultrasound. Radiology 1970;96:15-22.

7. Thoeni RF. The role of imaging in patients with ascites. AJR Am J Roentgenol 1995;165:16-8.

8. Nguyen PT, Chang KJ. EUS in the detection of ascites and EUS-guided paracentesis. Gastrointest Endosc 2001;54:336-9.

9. Kaushik N, Khalid A, Brody D, et al. EUS-guided paracentesis for the diagnosis of malignant ascites. Gastrointest Endosc 2006;64:908-13.

10. Alberghina N, Sanchez-Montes C, Tunon C, et al. Endoscopic ultrasonography can avoid unnecessary laparotomies in patients with pancreatic adenocarcinoma and undetected peritoneal carcinomatosis. Pancreatology 2017;17:858-864.

11. Hicks AM, Chou J, Capanu M, et al. Pancreas Adenocarcinoma: Ascites, Clinical Manifestations, and Management Implications. Clin Colorectal Cancer 2016;15:360-368.

12. Rana SS, Bhasin DK, Srinivasan R, et al. Endoscopic ultrasound-guided fine needle aspiration of peritoneal nodules in patients with ascites of unknown cause. Endoscopy 2011;43:1010-3.

13. Peter S, Eltoum I, Eloubeidi MA. EUS-guided FNA of peritoneal carcinomatosis in patients with unknown primary malignancy. Gastrointest Endosc 2009;70:1266-70.

14. Rana SS, Bhasin DK, Gupta R, et al. EUS-guided FNA of peritoneal carcinomatosis. Gastrointest Endosc 2011;73:188-9.

15. Becker G, Galandi D, Blum HE. Malignant ascites: systematic review and guideline for treatment. Eur J Cancer 2006;42:589-97.

16. DeWitt J, LeBlanc J, McHenry L, et al. Endoscopic ultrasound-guided fine-needle aspiration of ascites. Clin Gastroenterol Hepatol 2007;5:609-15.

CQ 4.

１ Allen VB, Gurusamy KS, Takwoingi Y, et al. Diagnostic accuracy of laparoscopy following computed tomography（CT）scanning for assessing the resectability with curative intent in pancreatic and periampullary cancer. Cochrane Database Syst Rev 2016；7：CD009323.

２　Ta R, O'Connor DB, Sulistijo A, et al. The role of staging laparoscopy in resectable and borderline resectable pancreatic cancer：a systematic review and meta-analysis. Dig Surg 2018; 36(3):251-260.

３　Hariharan D, Constantinides VA, Froeling FE, et al. The role of laparoscopy and laparoscopic ultrasound in the preoperative staging of pancreatico-biliary cancers--A meta-analysis. Eur J Surg Oncol 2010；36：941-8.

４　Satoi S, Yanagimoto H, Yamamoto T, et al. A clinical role of staging laparoscopy in patients with radiographically defined locally advanced pancreatic ductal adenocarcinoma. World J Surg Oncol 2016；14：14.

５　Clark CJ, Traverso LW. Positive peritoneal lavage cytology is a predictor of worse survival in locally advanced pancreatic cancer. Am J Surg. 2010;199:657–62.

６　Morak MJ, Hermans JJ, Smeenk HG, et al. Staging for locally advanced pancreatic cancer. Eur J Surg Oncol. 2009;35:963–8.

７ Shoup M, Winston C, Brennan MF, Bassman D, Conlon KC. Is there a role for staging laparoscopy in patients with locally advanced, unresectable pancreatic adenocarcinoma? J Gastrointest Surg. 2004;8:1068–71.

８ Contreras CM, Stanelle EJ, Mansour J, et al. Staging laparoscopy enhances the detection of occult metastases in patients with pancreatic adenocarcinoma. J Surg Oncol 2009；100：663-9.

９ Schnelldorfer T, Gagnon AI, Birkett RT, et al. Staging laparoscopy in pancreatic cancer：a potential role for advanced laparoscopic techniques. J Am Coll Surg 2014；218：1201-6.

１０ M. Suker, B. Groot Koerkamp, C.H.J. van Eijck, et al. Yield of staging laparoscopy before treatment of locally advanced pancreatic cancer to detect occult metastases. European Journal of Surgical Oncology 2019 ; 45: 1906-11.

１１ Peng JS, Mino J, Monteiro R, et al. Diagnostic laparoscopy prior to neoadjuvant therapy in pancreatic cancer is high yield：an analysis of outcomes and costs. J Gastrointest Surg 2017；21：1420-27.

12 T. Takadate, T. Morikawa, M. Unno, et al. Staging laparoscopy is mandatory for the treatment of pancreatic cancer to avoid missing radiologically negative metastases. Surgery Today 2020 Sep 8. doi: 10.1007/s00595-020-02121-4. Online ahead of print.

１3 De Rosa A, Cameron IC, Gomez D. Indications for staging laparoscopy in pancreatic cancer. HPB（Oxford）2016；18：13-20.

１4　Karabicak I, Satoi S, Yanagimoto H, et al. Risk factors for latent distant organ metastasis detected by staging laparoscopy in patients with radiologically defined locally advanced pancreatic ductal adenocarcinoma. J Hepatobiliary Pancreat Sci. 2016;23:750-755.

CQ 5.

1. Conroy T, Desseigne F, Ducreux M et al. ; Groupe Tumeurs Digestives of Unicancer; PRODIGE Intergroup. FOLFIRINOX versus gemcitabine for metastatic pancreatic cancer. N Engl J Med. 2011 May 12;364(19):1817-25.

2. Von Hoff DD, Ervin T, Renschler MF et al. Increased survival in pancreatic cancer with nab-paclitaxel plus gemcitabine. N Engl J Med. 2013 Oct 31;369(18):1691-703.

3. Ueno H, Ioka T, Tanaka M, et al. Randomized phase III study of gemcitabine plus S-1, S-1 alone, or gemcitabine alone in patients with locally advanced and metastatic pancreatic cancer in Japan and Taiwan: GEST study. J Clin Oncol. 2013 May 1;31(13):1640-8.

4. Moore MJ, Goldstein D, Parulekar W, et al.; National Cancer Institute of Canada Clinical Trials Group. Erlotinib plus gemcitabine compared with gemcitabine alone in patients with advanced pancreatic cancer: a phase III trial of the National Cancer Institute of Canada Clinical Trials Group. J Clin Oncol. 2007 May 20;25(15):1960-6.

5. Todaka A, Mizuno N, Fukutomi A, et al. Nationwide Multicenter Observational Study of FOLFIRINOX Chemotherapy in 399 Patients With Unresectable or Recurrent Pancreatic Cancer in Japan. Pancreas. 2018 May/Jun;47(5):631-636.

6. Thomassen I, Lemmens VE, de Hingh IH et al. Incidence, prognosis, and possible treatment strategies of peritoneal carcinomatosis of pancreatic origin: a population-based study. Pancreas. 2013 Jan;42(1):72-5.

7. Sasaki T, Kanata R, Sasahira N, et al. Improvement of Treatment Outcomes for Metastatic Pancreatic Cancer: A Real-world Data Analysis. In Vivo. 2019 Jan-Feb;33(1):271-276.

8. Takahara N, Isayama H, Koike K, et al. Pancreatic cancer with malignant ascites: clinical features and outcomes. Pancreas. 2015 Apr;44(3):380-5.

9. Bonnet E, Mastier C, de La Fouchardière C, et al. FOLFIRINOX in patients with peritoneal carcinomatosis from pancreatic adenocarcinoma: a retrospective study. Curr Oncol. 2019 Aug;26(4):e466-e472.

10. Satoi S, Yamaue H, Takada T, et al. Role of adjuvant surgery for patients with initially unresectable pancreatic cancer with a long-term favorable response to non-surgical anti-cancer treatments: results of a project study for pancreatic surgery by the Japanese Society of Hepato-Biliary-Pancreatic Surgery. J Hepatobiliary Pancreat Sci. 2013 Aug; 20(6):590-600.

11. Ichikawa Y, Yamada D, Doki Y, et al. A Case Report of Curative Surgery for Pancreatic Ductal Adenocarcinoma with Peritoneal Dissemination after Gemcitabine Chemotherapy. Gan To Kagaku Ryoho. 2017 Nov;44(12):2014-2016.

12. Kobayashi Y, Maeda S, Nakamori S, et al. Successful conversion surgery for unresectable pancreatic cancer with peritoneal metastases after neoadjuvant albumin-bound paclitaxel and gemcitabine chemotherapy: case report and literature review. Int Cancer Conf J. 2017 Nov 21;7(1):20-25.

13. Yoshii H, Izumi H, Makuuchi H, et al. GEM + nab-PTX Therapy for Pancreatic Body Cancer cStage IVb for Conversion Surgery: A Case Report. Tokai J Exp Clin Med. 2019 Dec 20;44(4):85-89.CQ5：腹膜播種を有する膵癌に対して全身化学療法を推奨するか？

CQ 6.

1. Takahara N, Isayama H, Nakai Y, Sasaki T, Saito K, Hamada T, Mizuno S, Miyabayashi K, Mohri D, Kogure H, Matsubara S, Yamamoto N, Hirano K, Ijichi H, Tateishi K, Tada M, Koike K. Pancreatic cancer with malignant ascites: clinical features and outcomes. Pancreas. 2015 Apr;44(3):380-5. doi: 10.1097/MPA.0000000000000290. PMID: 25636085
2. Takahara N, Isayama H, Nakai Y, Ishigami H, Satoi S, Mizuno S, Kogure H, Matsubara S, Yamamoto N, Yamaguchi H, Tada M, Kitayama J, Watanabe T, Koike K. Intravenous and intraperitoneal paclitaxel with S-1 for treatment of refractory pancreatic cancer with malignant ascites. Invest New Drugs. 2016 Oct;34(5):636-42. doi: 10.1007/s10637-016-0369-0. Epub 2016 Jun 23. PMID: 27339809l.
3. Satoi S, Yanagimoto H, Yamamoto T, Hirooka S, Yamaki S, Kosaka H, Inoue K, Hashimoto Y, Matsui Y, Kon M. Survival benefit of intravenous and intraperitoneal paclitaxel with S-1 in pancreatic ductal adenocarcinoma patients with peritoneal metastasis: a retrospective study in a single institution. J Hepatobiliary Pancreat Sci. 2017 May;24(5):289-296. doi: 10.1002/jhbp.447. Epub 2017 Apr 19. PMID: 28301088
4. Satoi S, Fujii T, Yanagimoto H, Motoi F, Kurata M, Takahara N, Yamada S, Yamamoto T, Mizuma M, Honda G, Isayama H, Unno M, Kodera Y, Ishigami H, Kon M. Multicenter Phase II Study of Intravenous and Intraperitoneal Paclitaxel With S-1 for Pancreatic Ductal Adenocarcinoma Patients With Peritoneal Metastasis. Ann Surg. 2017 Feb;265(2):397-401. doi: 10.1097/SLA.0000000000001705. PMID: 28059968 Clinical Trial.
5. Takahara N, Nakai Y, Ishigami H, Saito K, Sato T, Hakuta R, Ishigaki K, Saito T, Hamada T, Mizuno S, Kogure H, Yamashita H, Isayama H, Seto Y, Koike K. A phase I study of intraperitoneal paclitaxel combined with gemcitabine plus nab-paclitaxel for pancreatic cancer with peritoneal metastasis. Invest New Drugs. 2020 Aug 8. doi: 10.1007/s10637-020-00982-7. Epub ahead of print. PMID: 32772340.
6. Yamada S, Fujii T, Yamamoto T, Takami H, Yoshioka I, Yamaki S, Sonohara F, Shibuya K, Motoi F, Hirano S, Murakami Y, Inoue H, Hayashi M, Murotani K, Kitayama J, Ishikawa H, Kodera Y, Sekimoto M, Satoi S. Phase I/II study of adding intraperitoneal paclitaxel in patients with pancreatic cancer and peritoneal metastasis. Br J Surg. 2020 Jul 7. doi: 10.1002/bjs.11792. Epub ahead of print. PMID: 32638367.

CQ 7.

1. Farma JM, Pingpank JF, Libutti SK, Bartlett DL, Ohl S, Beresneva T et al. Limited survival in patients with carcinomatosis from foregut malignancies after cytoreduction and continuous hyperthermic peritoneal perfusion. J Gastrointest Surg. 2005;9(9):1346-53
2. Tentes AA, Pallas N, Karamveri C, Kyziridis D, Hristakis C. Cytoreduction and HIPEC for peritoneal carcinomatosis of pancreatic cancer. J BUON. 2018;23(2):482-7
3. Lin SD, Soucisse ML, Lansom J, Morris DL. Cytoreductive surgery and hyperthermic intraperitoneal chemotherapy in a patient with peritoneal carcinomatosis from a pancreatic cystadenocarcinoma: A case report. Int J Surg Case Rep. 2019;63:48-52

CQ 8.

1. Satoi S, Yanagimoto H, Yamamoto T, Toyokawa H, Hirooka S, Yamaki S, Opendro SS, Inoue K, Michiura T, Ryota H, Matsui Y, Kon M. A clinical role of staging laparoscopy in patients with radiographically defined locally advanced pancreatic ductal adenocarcinoma. World J Surg Oncol. 2016; 20;14:14.

2. Tsuchida H, Fujii T, Mizuma M, Satoi S, Igarashi H, Eguchi H, Kuroki T, Shimizu Y, Tani M, Tanno S, Tsuji Y, Hirooka Y, Masamune A, Mizumoto K, Itoi T, Egawa S, Kodama Y, Hamada S, Unno M, Yamaue H, Okazaki K; Committee of Clinical Research, Japan Pancreas Society. Prognostic importance of peritoneal washing cytology in patients with otherwise resectable pancreatic ductal adenocarcinoma who underwent pancreatectomy: A nationwide, cancer registry-based study from the Japan Pancreas Society. Surgery. 2019; 166:997-1003.

3. Satoi S, Fujii T, Yanagimoto H, Motoi F, Kurata M, Takahara N, Yamada S, Yamamoto T, Mizuma M, Honda G, Isayama H, Unno M, Kodera Y, Ishigami H, Kon M. Multicenter Phase II Study of Intravenous and Intraperitoneal Paclitaxel With S-1 for Pancreatic Ductal Adenocarcinoma Patients With Peritoneal Metastasis. Ann Surg. 2017;265:397-401.

4. Satoi S, Yanagimoto H, Yamamoto T, Hirooka S, Yamaki S, Kosaka H, Inoue K, Hashimoto Y, Matsui Y, Kon M. Survival benefit of intravenous and intraperitoneal paclitaxel with S-1 in pancreatic ductal adenocarcinoma patients with peritoneal metastasis: a retrospective study in a single institution. J Hepatobiliary Pancreat Sci. 2017;24:289-296.

5. Yamada S, Fujii T, Yamamoto T, Takami H, Yoshioka I, Yamaki S, Sonohara F, Shibuya K, Motoi F, Hirano S, Murakami Y, Inoue H, Hayashi M, Murotani K, Kitayama J, Ishikawa H, Kodera Y, Sekimoto M, Satoi S. Phase I/II study of adding intraperitoneal paclitaxel in patients with pancreatic cancer and peritoneal metastasis. Br J Surg. 2020 Jul 7. doi: 10.1002/bjs.11792.

6. 客本ゆき恵, 高舘達之, 水間正道, 畠達夫, 伊関雅裕, 大塚英郎, 坂田直昭, 中川圭, 森川孝則, 林洋毅, 元井冬彦, 内藤剛, 菅野敦, 下瀬川徹, 海野倫明. 腹膜転移を有する膵頭部癌に対して Adjuvant Surgery を企図し切除し得た1例. 癌と化学療法, 2017, 44.12: 1880-1882.

7. 木村寛伸, 伏田幸夫, 武川昭男. S-1/Gemcitabine, Paclitaxel 併用療法が奏効し根治手術が可能となった腹膜播種を伴う膵体部癌1例. 癌と化学療法, 2009, 36.7: 1191-1194.

8. 牧野裕庸, 亀高尚, 深田忠臣, 清家和裕, 小山隆史, 長谷川章雄. FOLFIRINOX 療法でDown-Stagingの後DP-CARにてR0切除を施行し得た膵癌の1例. 癌と化学療法, 2015, 42.12: 1644-1646.

CQ 9.

1. Yachida S, Fukushima N, Sakamoto M, Matsuno Y, Kosuge T, Hirohashi S. Implications of peritoneal washing cytology in patients with potentially resectable pancreatic cancer. Br J Surg. 2002;89(5):573-578.

2. Ferrone CR, Haas B, Tang L, et al. The influence of positive peritoneal cytology on survival in patients with pancreatic adenocarcinoma. J Gastrointest Surg. 2006;10(10):1347-1353.

3. Yamada S, Takeda S, Fujii T, et al. Clinical implications of peritoneal cytology in potentially resectable pancreatic cancer: positive peritoneal cytology may not confer an adverse prognosis. Ann Surg. 2007;246(2):254-258.

4. Konishi M, Kinoshita T, Nakagohri T, Inoue K, Oda T, Takahashi S. Prognostic value of cytologic examination of peritoneal washings in pancreatic cancer. Archives of surgery (Chicago, Ill : 1960). 2002;137(4):475-480.

5. Yamada S, Fujii T, Kanda M, et al. Value of peritoneal cytology in potentially resectable pancreatic cancer. Br J Surg. 2013;100(13):1791-1796.

6. Abe T, Ohuchida K, Endo S, et al. Clinical importance of intraoperative peritoneal cytology in patients with pancreatic cancer. Surgery. 2017;161(4):951-958.

7. Satoi S, Murakami Y, Motoi F, et al. Reappraisal of peritoneal washing cytology in 984 patients with pancreatic ductal adenocarcinoma who underwent margin-negative resection. J Gastrointest Surg. 2015;19(1):6-14; discussion 14.

8. Yoshioka R, Saiura A, Koga R, et al. The implications of positive peritoneal lavage cytology in potentially resectable pancreatic cancer. World J Surg. 2012;36(9):2187-2191.

9. Hoshimoto S, Hishinuma S, Shirakawa H, et al. Prognostic significance of intraoperative peritoneal washing cytology for patients with potentially resectable pancreatic ductal adenocarcinoma. Pancreatology. 2017;17(1):109-114.

10. Hirabayashi K, Imoto A, Yamada M, et al. Positive Intraoperative Peritoneal Lavage Cytology is a Negative Prognostic Factor in Pancreatic Ductal Adenocarcinoma: A Retrospective Single-Center Study. Front Oncol. 2015;5:182.

11. Steen W, Blom R, Busch O, et al. Prognostic value of occult tumor cells obtained by peritoneal lavage in patients with resectable pancreatic cancer and no ascites: A systematic review. J Surg Oncol. 2016;114(6):743-751.

12. Cao F, Li J, Li A, Li F. Prognostic significance of positive peritoneal cytology in resectable pancreatic cancer: a systemic review and meta-analysis. Oncotarget. 2017;8(9):15004-15013.

13. Tsuchida H, Fujii T, Mizuma M, et al. Prognostic importance of peritoneal washing cytology in patients with otherwise resectable pancreatic ductal adenocarcinoma who underwent pancreatectomy: A nationwide, cancer registry-based study from the Japan Pancreas Society. Surgery. 2019;166(6):997-1003.

Column

1. Solass W, Kerb R, Mürdter T, Giger-Pabst U, Strumberg D, Tempfer C, Zieren J, Schwab M, Reymond MA. Intraperitoneal chemotherapy of peritoneal carcinomatosis using pressurized aerosol as an alternative to liquid solution: first evidence for efficacy. Ann Surg Oncol. 2014 Feb;21(2):553-9.

2. Grass F, Vuagniaux A, Teixeira-Farinha H, Lehmann K, Demartines N, Hübner M. Systematic review of pressurized intraperitoneal aerosol chemotherapy for the treatment of advanced peritoneal carcinomatosis. Br J Surg. 2017 May;104(6):669-678.

3. Alyami M, Hübner M, Grass F, Bakrin N, Villeneuve L, Laplace N, Passot G, Glehen O, Kepenekian V. Pressurised intraperitoneal aerosol chemotherapy: rationale, evidence, and potential indications. Lancet Oncol. 2019 Jul;20(7):e368-e377.

4. Graversen M, Detlefsen S, Bjerregaard JK, Pfeiffer P, Mortensen MB. Peritoneal metastasis from pancreatic cancer treated with pressurized intraperitoneal aerosol chemotherapy (PIPAC). Clin Exp Metastasis. 2017 Jun;34(5):309-314.

5. Horvath P, Beckert S, Struller F, Königsrainer A, Reymond MA. Pressurized intraperitoneal aerosol chemotherapy (PIPAC) for peritoneal metastases of pancreas and biliary tract cancer. Clin Exp Metastasis. 2018 Oct;35(7):635-640.

6. Di Giorgio A, Sgarbura O, Rotolo S, Schena CA, Bagalà C, Inzani F, Russo A, Chiantera V, Pacelli F. Pressurized intraperitoneal aerosol chemotherapy with cisplatin and doxorubicin or oxaliplatin for peritoneal metastasis from pancreatic adenocarcinoma and cholangiocarcinoma. Ther Adv Med Oncol. 2020 Jul 24;12:1758835920940887.

7. Nielsen M, Graversen M, Ellebæk SB, Kristensen TK, Fristrup C, Pfeiffer P, Mortensen MB, Detlefsen S. Next-generation sequencing and histological response assessment in peritoneal metastasis from pancreatic cancer treated with PIPAC. JClin Pathol. 2020 May 8:jclinpath-2020-206607.
